# Seeking consent to genetic and genomic research in a rural Ghanaian setting: A qualitative study of the MalariaGEN experience

**DOI:** 10.1186/1472-6939-13-15

**Published:** 2012-07-02

**Authors:** Paulina Tindana, Susan Bull, Lucas Amenga-Etego, Jantina de Vries, Raymond Aborigo, Kwadwo Koram, Dominic Kwiatkowski, Michael Parker

**Affiliations:** 1 The Ethox Centre, Department of Public Health, University of Oxford, Old Road Campus, Headington, Oxford, OX3 7LF, United Kingdom; 2 Navrongo Health Research Centre, Ghana Health Service, P.O.Box 114, Navrongo, Ghana; 3 MRC Centre for Genomics and Global Health, University of Oxford, Oxford, OX3 7BN, United Kingdom; 4 Noguchi Memorial Institute for Medical Research, University of Ghana, Legon, Ghana; 5 Wellcome Trust Sanger Institute, Hinxton, Cambridge, CB10 1SA, United Kingdom

**Keywords:** Consent, Genetic research, Genomic research, Research ethics, Qualitative research, Ghana, Africa

## Abstract

**Background:**

Seeking consent for genetic and genomic research can be challenging, particularly in populations with low literacy levels, and in emergency situations. All of these factors were relevant to the MalariaGEN study of genetic factors influencing immune responses to malaria in northern rural Ghana. This study sought to identify issues arising in practice during the enrolment of paediatric cases with severe malaria and matched healthy controls into the MalariaGEN study.

**Methods:**

The study used a rapid assessment incorporating multiple qualitative methods including in depth interviews, focus group discussions and observations of consent processes. Differences between verbal information provided during community engagement processes, and consent processes during the enrolment of cases and controls were identified, as well as the factors influencing the tailoring of such information.

**Results:**

MalariaGEN participants and field staff seeking consent were generally satisfied with their understanding of the project and were familiar with aspects of the study relating to malaria. Some genetic aspects of the study were also well understood. Participants and staff seeking consent were less aware of the methodologies employed during genomic research and their implications, such as the breadth of data generated and the potential for future secondary research.

Moreover, trust in and previous experience with the Navrongo Health Research Centre which was conducting the research influenced beliefs about the benefits of participating in the MalariaGEN study and subsequent decision-making about research participation.

**Conclusions:**

It is important to recognise that some aspects of complex genomic research may be of less interest to and less well understood by research participants and that such gaps in understanding may not be entirely addressed by best practice in the design and conduct of consent processes. In such circumstances consideration needs to be given to additional protections for participants that may need to be implemented in such research, and how best to provide such protections.

Capacity building for research ethics committees with limited familiarity with genetic and genomic research, and appropriate engagement with communities to elicit opinions of the ethical issues arising and acceptability of downstream uses of genome wide association data are likely to be important.

## Background

The need for voluntary and informed consent to research is enshrined in international guidance and regulation on research ethics [1,2]. Consent processes for research serve two purposes: first they are a means of respecting participants’ autonomy and decision-making capacity. Secondly, they can protect prospective participants by providing them with information about potential harms of research, and enabling them to choose to avoid these by declining to take part in research.

Empirical research demonstrates that researchers encounter numerous challenges in practice when seeking to implement consent processes that support potential research participants in making voluntary and informed decisions [3,4]. Particular challenges can arise when seeking consent from impoverished populations with high levels of illiteracy [5-7], when seeking consent to paediatric research [8], when seeking consent to research in emergency situations [9-11] and when seeking consent to genomic research [12]. Specific challenges arising when seeking consent for genetic and genomic research include explaining the research methods, the implications of the complex informatics infrastructure required to support such studies and the potential consequences of future research with samples and data (the nature of which may be unknown at the time consent is sought)[13].

All of these factors were relevant in the recruitment of participants into a case–control study to identify genetic factors affecting immune responses to malaria under the auspices of the MalariaGEN consortium [14]. In this study the cases were children admitted to the Navrongo War Memorial Hospital with diagnosis of severe malaria; and the controls were drawn from healthy children in the cases’ communities.

The MalariaGEN network incorporates large-scale genome-wide association (GWA) studies to identify genetic variants that are associated with resistance or susceptibility to severe malaria [15].

It compares genetic markers throughout the genomes of patients with malaria (cases) and of healthy individuals from the same populations (controls) to look for differences between these groups that correlate with resistance to disease [16]. This project has study sites in 11 malaria–endemic countries, including two sites in Ghana. Data from individual study sites are used for genome-wide analyses or for studying selected genetic variants. MalariaGEN releases the GWA data it generates to external researchers for further analyses. To facilitate this process it has developed a data release policy and data are released via an Independent Data Access Committee [17].

There is a limited but growing literature on seeking informed consent to research in developing countries and a developing discourse on informed consent and genomic research in developed countries [18-20]. In response to challenges arising when seeking consent to genomic research MalariaGEN developed a template and guidelines for obtaining informed consent in consultation with researchers and the ethics review committees reviewing the study [21]. These materials were designed to balance the need for fundamental human subject protections to be preserved across all of the malaria-endemic sites where participants were being recruited, while still allowing for appropriate tailoring of consent processes to each research context.

We are aware of very few published empirical studies on seeking consent for genetic or genomic research in Africa [22,24]. The increasing interest in conducting genomic research in Africa, such as that to be funded by the Human Heredity and Health in Africa (H3 Africa) initiative, suggests that such research is urgently needed.

The aim of our study was to examine the issues arising when enrolling participants into a MalariaGEN project in the Kassena-Nankana District (KND) in northern Ghana, using consent materials adapted from the MalariaGEN guidelines. Previous studies conducted in the KND on informed consent and community engagement [5,25] have described the role of traditional decision-making structures in the consent process, the role of trust in sustaining researcher–participant relationship and some of the constraints of contextualizing current international consent requirements. The focus of this study was to provide insights into specific issues arising when seeking consent to genetic and genomic research in a low-income context, to identify examples of best practice in enrolling participants in this context, and to identify areas that may need further research and discussion in this rural setting.

Rapid assessment methods were used to review the initial design and conduct of the consent processes. Ways in which consent processes were amended as researchers and fieldworkers sought to achieve best practice in enrolling participants were reviewed and participants’ experiences and views of the research were also sought. This paper concludes by discussing the potential limitations of consent as a mechanism for legitimising genomic research in a low income setting and the additional measures to respect research participants that it may be appropriate to implement in future genetic and genomic studies in such contexts.

## Methods

Rapid assessments are an established tool within the social sciences designed to inform ‘the adaptation of interventions to local cultures and conditions’ [26]. Assessments are informed by an intensive data collection exercise which employs multiple qualitative methods - such as participant observation, semi-structured interviews, focus group discussions and collection of unpublished data. Data are then rapidly and iteratively analysed, and recommendations made. Information gained from rapid assessments has been proven valuable in other health-related contexts in Africa [27] and calls have been made for such assessments to be routinely conducted to inform the design of consent processes before conducting trials in developing countries [28,29]. Researchers have recently demonstrated the value of using a rapid assessment to inform the design of a consent process for genetic research in Ethiopia [23,24].

### Study site

This study was conducted in the KND, one of the administrative districts of northern Ghana and the location of the Navrongo Health Research Centre (NHRC). KND covers a land area of 1,675 km2 and has an estimated population of 151,000. The two main ethnic groups that live in the district are the Kassenas and Nankanis. Both groups share a traditional rural agrarian culture and traditions but speak different languages. In terms of traditional jurisdiction the KND is divided into Chiefdoms, headed by a male Chief and a council of male elders representing various communities. Within these traditional boundaries, the Kassenas and Nankanis are grouped into compounds, which are usually headed by the most elderly male. Compounds are then further divided into households, in which multiple generations of a family typically reside. The current literacy rate in the KND is 59%.

The Navrongo Health Research Centre (NHRC) is based in the KND and was established during a Vitamin A supplementation trial (VAST), which was conducted in 1989. The Centre is still colloquially known as ‘VAST’ rather than NHRC in the community.

The NHRC has also conducted research into diseases such as malaria, HIV/AIDS, human rotavirus diarrhoea, meningitis and lymphatic filariasis. The findings from a number of NHRC trials, including the Vitamin A supplementation trial, have influenced health service provision in Ghana and internationally [30,31].

The NHRC contributes to MalariaGEN in collaboration with the Noguchi Memorial Institute for Medical Research in Accra, Ghana.

### The MalariaGEN consent process

**Figure 1:**
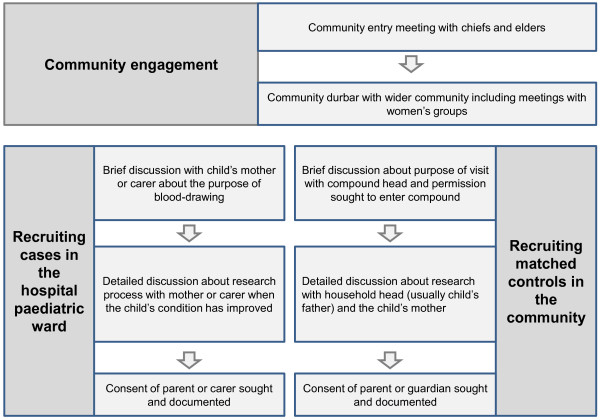
Providing information to potential participants.

During the MalariaGEN study, information was provided to prospective participants in three different ways. These were a community engagement process prior to the initiation of the study, a two-stage enrolment process for mothers or caregivers of cases (children with severe malaria who presented at the hospital) and an enrolment process at the community level for matched controls (as illustrated in Figure 1).

### Data collection

For this qualitative study, data were collected for six weeks over a six month period, beginning in September 2008. Data collection included face-to-face semi-structured interviews, focus group discussions (FGDs) and observation of consent processes for the MalariaGEN project. Information sheets, consent forms, community entry statements and videos of community meetings about the study were also reviewed.

The majority of the data analysed in this study came from interviews and FGDs with research scientists, research assistants, fieldworkers and parents of research participants, as outlined below. The content of the interviews and FGDs was directed by a field guide that addressed knowledge and understanding of the MalariaGEN study, the consent process, voluntariness and motivation for participation in research and future uses of samples. Thirteen interviews were conducted in English while eight interviews and the six FGDs were conducted in the local languages of the participants.

To corroborate and expand on information gained through the semi-structured interviews, a small number of consent processes for enrolling cases and controls were observed. The observers generated detailed field notes and a debriefing session was later held with the participants to discuss their experiences of the consent process.

This study was approved by the Oxford Tropical Research Ethics Committee of the University of Oxford, UK (ref 15 08) and Navrongo Health Research Centre’s Institutional Review Board, Navrongo, Ghana (ref NHRCIRC074).

### Sampling

The study incorporated purposive sampling of all the key informants involved in the MalariaGEN project. The MalariaGEN data fellow, the three clinicians caring for study participants, an additional NHRC researcher, two research assistants and four project staff at the hospital recruiting cases and two fieldworkers recruiting controls were interviewed. A sample of research participants (mothers of cases or controls enrolled in the study within the previous three months) were contacted sequentially and invited to be interviewed or attend focus groups.

Written consent, which was preceded by a verbal explanation of the study objectives and procedures, was obtained from all participants in the study. The forms were also translated into the two major local languages [Kasem and Nankam] for non-English speaking participants, particularly the mothers of the cases and controls. A total of 84 individuals participated in this study: 24 in face-to-face semi-structured interviews and ten participants in each of the six focus group discussions (see Table 1).

**Table 1:** Research participants

**Category**	**Participants**
Research scientists (male)	5 (interviews)
Research Assistants (male)	2 (interviews)
Research staff (2 male fieldworkers recruiting controls and 4 female project staff recruiting cases in the wards)	6 (interviews)
Mothers of cases	34 (4 interviews and 3 focus groups with 10 per group)
Mothers of controls	34 (4 interviews and 3 focus groups with 10 per group)
**Total number of participants**	**84**

### Data analysis

Data analysis was conducted iteratively throughout the study. During the data collection period, preliminary analysis of the initial interviews took place in KND and the issues raised were further explored in subsequent data collection. Interviews and FGDs conducted in local languages were translated into English. All interviews were transcribed, anonymised and imported into qualitative data analysis software (NVivo 8) for further analysis.

## Results

### The consent process

To obtain meaningful informed consent from study participants requires the provision of information in a manner that enhances participants’ understanding and appreciation of relevant aspects of proposed research. Having a well-written consent form does not guarantee participants’ understanding, particularly in populations with underdeveloped local languages and high levels of illiteracy which rely on verbal explanations about research. While the consent forms used for the MalariaGEN study outlined all the salient aspects of the research, the research team’s views about what information was most relevant to the intended audience determined what information was provided verbally at these three stages. Broadly speaking, during the community engagement processes and the enrolment of controls at the community level there was more space for discussions about genomic aspects of the research than during recruitment of cases in the ward.

### Engaging local communities in genomic research

In KND, the NHRC has established a model of engaging local communities in research based on the traditional practices of the Kassena-Nankana community [32]. As reported by previous studies conducted in the KND [5,25], traditionally, consultation with the gatekeepers of the community (chiefs and elders) is required before any research is carried out in the community, followed by community durbars (meetings) with the wider community. The MalariaGEN study utilised these existing engagement processes.

"*We first of all went to talk to the chiefs and then told them that this study is going to be conducted and people would enter the community to talk to community members and pick samples and … when a child gets sick and comes to the ward and it’s severe malaria disease this is what we are going to do [we tried] to explain the study to them. (Research Assistant)*"

The MalariaGEN research team reported that this community entry process aimed to seek permission from gatekeepers of the community to conduct the study and to inform the wider community about the proposed research and its implications. The team recognised that the standard community entry process had limitations because women were infrequently involved in the process but had an important role in consenting to research involving young children in emergency situations (which frequently involve severe malaria in this context) [25].

Consequently they went a step further than is usual during community engagement exercises for NHRC studies and organised meetings with women’s groups to discuss the MalariaGEN study with them. In total five large community durbars were held in addition to seven smaller meetings with women’s groups.

During community meetings discussions covered the purpose of the study, the procedures, who would be involved and the expected outcomes of the study. Great care was taken to explain scientific aspects of the research and to translate complex information about topics such as DNA and genes into lay local languages with appropriate analogies to facilitate understanding.

"*…the thing in the blood that makes us different from one another and can identify people from the same family (Lay translation of genes during community engagement)*"

Identifying research populations for community engagement in advance for studies recruiting patients on admission to hospital can be challenging because of the size of the catchment areas for such facilities. In such contexts community engagement strategies such as the durbar have limitations in terms of providing information to prospective participants about the research in advance. However the discussions at the end of the meetings reassured researchers that the project was broadly acceptable to the community and that aspects of genetic and genomic research could be adequately explained to the population. Moreover preparing for, and reviewing the effectiveness of the provision of information in community engagement meetings can provide a valuable means of rehearsing effective provision of complex information during individual consent processes.

"*When we are doing the community meetings, that’s when we give more information, we try to explain the science aspects of these things, because you have the time to explain and explain and explain. (Researcher)*"

"*The experience started with the community entry when we tried to explain [the scientific terms]. The chiefs would be laughing and then they try to give [us] the appropriate word. (Fieldworker recruiting controls)*"

### Enrolling cases in the hospital

The consent process for recruiting cases (children with severe malaria) into the MalariaGEN study typically involved just the primary carer (usually the mother) who brought a child with severe malaria to the hospital. Discussions about the study were held at least twice. On admission to the hospital, mothers were asked if blood samples could be taken for both routine tests and potentially for research into severe malaria. A single blood draw was made for the purpose of clinical tests and DNA extraction for the research.

A more detailed discussion about the study was held subsequently if the child was eligible to participate. Subsequent to that discussion, if the child’s parent agreed, documentation for enrolment in the study would be completed. Blood for patients who did not meet the research criteria or whose primary care giver declined consent was only used for clinical purposes.

"*[The nurses] can't just start taking the sample without telling the mother what we are going to do or what we are about. So from there we give some small summary ‘Excuse me I want to take some little blood from your kid and I will talk to you later… and let you know more about what is in the blood’. (Project staff recruiting cases)*"

"*…when I went there with my child, it was in the morning so the nurses and doctor attended to my child. At that time the VAST workers were also there so they all helped to set up the water before they came and took the blood to go and test. (Focus group with mothers of cases)*"

The research team highlighted the complexities of seeking consent in hospital settings and especially in emergency situations. When children are admitted with severe malaria the priority is to provide appropriate treatment as rapidly as possible. After recruiting the initial cases researchers considered that it was practically impossible and ethically inappropriate to conduct a detailed consent process for research before collecting the samples needed for diagnosis and treatment. Consequently the research team needed to establish how to seek consent in a way that could maximize understanding and free decision-making.

The revised process in place during the rapid assessment involved project staff in charge of recruitment in the paediatric ward observing the condition of the sick child and the emotional state of the mother to ascertain an appropriate time to initiate the consent process. Consent to research was usually sought within 24 to 36 hours of admission. To protect privacy, mothers were invited to a private room for the discussion, although some preferred to discuss the research in the ward next to their child’s bed.

"*When the child is brought … [with] the convulsion the child is weak, cannot open his eyes, the mother will be weeping, walking up and down, but by the time they give the drugs, and the child can even talk or respond to the mother, then she feels better, we can then do our consenting. (Project staff recruiting cases)*"

There was consensus amongst the mothers interviewed that it was appropriate in the circumstances for a discussion of the project to take place after the blood samples were collected and their child’s condition had stabilised.

"*They should take the blood first and when the child gets better before they come to have the discussions with [me]. (Mother of case)*"

"*In my case it was after my child felt better the following day before they came and had the discussions with me. If the girl [project staff] had come on the day I brought the child I could not have listened to her. (Mother of case)*"

While the parents we interviewed were generally satisfied with the timing of the consent process, some mothers noted that they remained anxious about their children when discussions about the project took place.

"*When she was discussing with me my attention was there but my child’s illness was also on my mind. (Mother of case)*"

Although the consent form covered all the salient aspects of the research including the genetic component, the focus of the discussions during the consent process was the purpose of the study with an emphasis on the rationale for blood-taking. Discussions about malaria genetics were phrased in general terms: ‘the thing in the blood that makes your child sick and other children of the same age group not sick’.

"*Then we’ll tell her that we will go to her area to take about two children of the same age and same sex, everything. Those two children must be healthy as well, then we’ll test to [confirm] whether those children are having this sickness. If it happens that they are not, then we will try to know why they’re in the same area and this one has [malaria] while that one is not having it. (Project staff recruiting cases)*"

### Enrolling controls in the community

Following enrolment of a case, fieldworkers visited the case’s community to enrol a matched ‘healthy’ control (a child whose parents answered ‘Yes’ to the question ‘Is your child well today?’). Enrolling controls involved a traditional multi-layered process where the permission of the head of the compound as well as the head of the household was sought before parents were approached. Fieldworkers noted that it was important for all levels of authority to understand the selection criteria for controls and appreciate why a particular child in their compound was selected.

"*Traditionally, around here, before you go into somebody’s house you have to greet, so what we do is … seek permission to enter into the house from the compound head. So you tell theperson of your mission, who you are, where you are coming from and what jobs you want to do in his compound, who you are looking for, let that person understand how you got thatperson’s name. (Fieldworker recruiting controls)*"

Discussions about purpose of the visit with compound heads ranged from brief to lengthy, depending on the compound head’s interest in the research. Detailed discussions about the research were then held with household heads and parents. In contrast to cases in the hospital ward, parents of controls often had more leisure to consider participation and wanted more detail about why researchers sought to enrol one of their healthy children in a study. In such circumstances fieldworkers discussed the scientific rationale of the research in further detail.

"*Researchers feel that probably it is something to do with human genes because probably you have two people living in the same area… one is always sick with malaria but the other isn’t sick with malaria. When you take them to the laboratory to have a malaria test you might find out that the one that is not sick with malaria has got the malaria parasite, more than one who’s always ill. That means that it is something to do with our genes. Probably the other person's DNA or gene is different from yours, maybe it is stronger, so that fights the malaria though he has the parasite. (Fieldworker recruiting controls)*"

Data from fieldworkers illustrated that an important issue when enrolling controls was explaining why blood samples from healthy children were being sought.

"*What is unusual is that we go to take [blood from] the controls and not from a sick child but from a healthy child and we are taking blood samples because when your child is sick and you are taking blood you know you are coming for help but my child is not sick and you are coming for blood it is a problem! (Fieldworker recruiting controls)*"

Fieldworkers explained that an extensive explanation of the rationale for taking these samples often helped to allay concerns parents might have about this process and refusals to take part in the research were rare.

### Knowledge and understandings of genetic and genomic research

Findings from this study demonstrated that in addition to senior researchers and research assistants; project staff, fieldworkers and parents of cases and controls had a good level of comprehension of aspects of the MalariaGEN project relating to malaria, which is a familiar disease in KND.

In contrast, levels of understanding of the complex and unfamiliar topic of genetic research varied considerably amongst staff and participants. Fieldworkers enrolling controls, research assistants and researchers were familiar with the methods used in the study to identify genetic differences between cases and controls which may correlate to susceptibility or resistance to severe malaria. Project staff recruiting cases, and mothers of cases and controls demonstrated less understanding of the methods used in the study.

"*They said if I got home, they would visit our compound to get other three children and my child, take their blood sample and my child’s blood sample to compare and see whether their malaria is the same or not. If it is not the same, they have to know why my child’s and theirs is not the same. I don’t understand that aspect of it. (Focus group with mothers of cases)*"

Additionally, the research team clearly recognised the difficulty in explaining genomics in local languages and the need to identify innovative ways of explaining the 'essential characteristics' of the genomic research to participants. In general, how much of the science to communicate, given these limitations, remained a challenge at all stages of the engagement process.

"*It is very challenging because, the language is pretty much undeveloped, there is no… terminology for those things, you need to explain them… if I were to write how I would explain a gene [ina local language], it is probably like a page, just for one word. (Researcher)*"

"*We don’t have a term, a single word that we can use to say genes, and so you talk about the blood and then you talk about the individual differences, we say that there are certain thingsin your blood that bring about our individual differences and that is what we want to extract. So with this they actually are able to understand. (Research Assistant)*"

Mothers were aware that the MalariaGEN project was aimed at gaining knowledge about malaria to facilitate the discovery of effective treatment. They understood that the project method involved recruiting severely ill children at the hospital as well as healthy children in the community. Mothers understood the rationale of the MalariaGEN project in terms of looking at the children’s blood to find differences which could explain why some children got severe malaria and others did not. However, they did not engage with the scientific methods by which this would be done, and the implications of that are discussed in the following section.

"*When they were recruiting us for the study they told me my child has too many parasites and that a team from VAST [NHRC] was coming to conduct a study on malaria so I can decide to be part… they said also that, they will send down some people to come to our community, recruit a child who doesn’t have the malaria and repeat the same process to find out why that child doesn’t have and my child has. (Mother of case)*"

"*You know there is a kind of malaria that can attack a child and make him collapse; he did not see that happen to our children so he wanted to know why that did not happen to our children. (Mother of control)*"

Most mothers could understand a discussion about the genetics of malaria because they related genetics to heredity based on their experiences of diseases that run in families. However, extending this understanding to the broader concept of genomics was more challenging. It was particularly difficult to extrapolate mothers’ knowledge of heredity to explain genomics where such research involves population level sampling that does not necessarily involve families affected by the disease under study.

### Sample use and data sharing

One consequence of participants’ understanding the methods of a genomic study in general terms is the difficulty of explaining potential downstream consequences of data sharing and analysis. Researchers and fieldworkers discussed the complexities of explaining the rationale for multiple uses of samples and data, a common feature of genomic research:

"*The other difficulty is when you want to talk about data access. Data access in a community that does not appreciate computers…we have to have innovative ways of doing that. You know one time I was talking about data access in the durbar and then the question that came up was is it wireless, is it like TV? (Researcher)*"

The MalariaGEN Network has developed a data release policy in conjunction with the ethics committees governing research in communities donating samples [17]. This policy provides for controlled release of genomic data to legitimate external researchers for acceptable research purposes. In this study we attempted to explore issues relating to the acceptability of future uses of data. Given that the participants were not familiar with scientific methods involved in the research, discussions about future research uses could not focus on genomic data. Nonetheless some exploration of this topic was considered desirable.

During some of the FGDs and interviews questions were couched in broad terms and focussed on a more familiar and related topic of secondary sample use, such as: ‘When they use the blood for a study there may be some left over and an idea could come up for a new study. Would you say they should ask you before using the left over blood for the new study or should they continue working with it?’. Participants indicated that researchers could conduct ongoing studies with left over samples without seeking additional permission from sample donors.

"*I think such an idea is not bad because they want to research into finding new solutions to solve our health problems. (Focus group with mothers of cases)*"

"*If they will be able to research into new solutions, it will be beneficial to us. So we have no problem with that. (Focus group with mothers of cases)*"

In addition, some participants asked that they be contacted if relevant findings were generated, a topic considered further below in the discussion about boundaries between research and therapy.

"*To me when there is a leftover they can use it if in future there is a study, but they should get me informed later maybe to tell me the results. (Mother of case)*"

### Boundaries between research and therapy

An additional factor impacting on participants’ understandings of the MalariaGEN study and their decisions about participation were beliefs about the benefits of study participation and the widespread conception that research studies are for the benefit of participants. There is a wealth of literature on this latter belief, sometimes termed ‘therapeutic misconception’ which has been demonstrated in many countries [7,33-35] and highlighted as a concern in genomic research [36].

In most clinical trials conducted by the NHRC, research participants have access to free health care services for problems unrelated to the research and medical bills of research participants are often covered by the research team. The community is familiar with this process.

"*I have another child who ever participated in one of their studies; it was they who took care of the child to be healthy. I know how good it is. (Focus group with mothers of cases)*"

Consequently, although in the MalariaGEN study research participants’ parents were not informed prior to consenting that their hospital costs would be met if they took part in the project, study responses revealed that participants’ unmet needs and parents’ expectations of free medical care for their children were important motivating factors for participation. Some mothers’ phrasing suggested that although they had been told research participation was optional; in practice the benefits of research participation were perceived to be so great that it would be hard to decline the study, a feature also reported in other African contexts [7,37,38].

"*When the [project staff member] came he did not force us; he said whoever wanted could participate in it. I saw that it was beneficial; there are so many diseases and since they came to help us (the community), you had to agree … (Mother of control)*"

"*She said it was not compulsory, if you did not want to participate, you could refuse; I wanted to participate that was why I thumb printed. This is one of the papers, she said it was voluntary and I knew it would be beneficial to me. (Mother of case)*"

Comments from these mothers consistently suggested that they didn’t clearly distinguish between the different research projects conducted by the NHRC (or VAST as it is colloquially known), or where such distinctions are made, different types of studies are perceived to offer similar benefits, such as free healthcare for a certain period of time during the study.

"*My child always falls sick and anytime I send him there he was treated free under VAST. That is why I agreed to participate in this study too. (Mother of case)*"

Unlike some studies where it is not clear that participants are aware that there is a distinction between research and therapy, [34] in KND the NHRC is well known and parents of cases clearly understood they were participating in research and could distinguish between project staff and hospital staff.

"*The nurses wear white and blue; VAST workers wear mufti; that is how we know these are nurses and these are VAST workers (Focus group with mothers of cases)*"

"*I know where the VAST workers sit, where the nurses sit and even the doctor’s room. (Focus group with mothers of cases).*"

Although clearly distinguishing between research and healthcare staff, mothers of cases and controls were generally of the view that the work of NHRC is to help them address their family’s health needs and therefore it is beneficial to participate in the research.

"*I said if it was something that was not beneficial to our community, they would not have done it and since he wanted to recruit our children into the study, it would be beneficial to them. (Focus group with mothers of controls)*"

"*It was because malaria attacked other children around but did not attack my child; that was why I agreed so that the disease would not worry my child. (Mother of control)*"

This study demonstrates that participants’ views of NHRC, and their expectations of the benefits of research (based on their previous experiences NHRC and not just information provided to them during the consent process for the MalariaGEN study) are important factors in their decision-making. Consequently, even studies that do not offer direct benefits to participants, such as the MalariaGEN study, may be perceived as offering such benefits.

## Discussion

The complexity of genomic research has meant that in one such study participants were considered to require a Masters qualification in genetics or equivalent before having the capacity to properly appreciate the risks of being involved and to give informed consent [39]. In African genomic studies incorporating large population groups and disease cohorts the majority of participants involved are unlikely to have a substantial background knowledge of genetics and their understanding of the research will necessarily be affected [22,24]. In contexts such as KND in northern Ghana, populations may also have relatively low literacy rates and limited familiarity with many of the concepts involved in biomedical research and the information technology required for the management of genomic data. Nyika cautions against exaggerating the impact of illiteracy on understanding information in developing countries about research and urges researchers to make extra efforts to address the challenges of seeking consent to genetic and genomic research in developing countries [40].

Determining best practice in seeking consent given the constraints faced in various research contexts will always be a challenge for researchers. In the KND, previous studies [5,25] have reported some of these constraints which relate to enhancing general understanding of research. Our current study indicates that in the context of genetic and genomic research, which involve complex scientific research methodologies, the consent process can be further complicated by difficulties with making the scientific concepts comprehensible to staff seeking consent as well as to research participants.

NHRC researchers noted that explanations of genomic aspects of research are likely to be particularly time consuming for participants, given literacy levels and the limitations of local languages for explaining such concepts, a factor commented on by researchers in other developing country contexts [36,41].

Another constraint highlighted in this study is the conduct of research in emergency situations which may make standard consent processes impracticable. The findings from this study provide examples of researchers’ responses to such constraints such as the care taken during community engagement exercises to develop ways of explaining genomic concepts and incorporating a two stage consent process to facilitate understanding in emergency situations. This study also identified factors that could improve understanding in future genomic research. One example is providing additional specialised training to research staff seeking consent for such a complex topic and ensuring that the lessons learned by such staff are communicated to those seeking consent for future genomic studies.

Variations in knowledge and understanding of genomics amongst staff recruiting participants in the MalariaGEN study could be attributed to the training they have received on this topic and their prior experience of other genetic studies. Both project staff on the wards enrolling cases and fieldworkers enrolling controls had secondary education and experience of enrolling participants in other case–control and genetic studies prior to the MalariaGEN study. The training they received for enrolling participants in this MalariaGEN study included exercises on explaining the genetic aspect of the research. In contrast to project staff on the wards, fieldworkers not only received additional training about genetic aspects of research as a consequence of recruiting participants into more than one MalariaGEN study, they also had more opportunity to rehearse the provision of information on these topics during the consent processes for healthy controls at the community level and during community engagement exercises.

More than one factor may have contributed to the fact project staff did not discuss genetics in detail when explaining the rationale of the research to cases’ mothers. One consideration is that they did not receive the additional training sessions about genetic aspects of research that fieldworkers did and may not have felt as comfortable explaining the research methods as a result. Additionally project staff were very aware that parents of cases on the ward were still under some stress and did not want to spend a disproportionate amount of time on consent, so they focused on topics considered of most importance to parents, such as the purposes of blood-taking.

Our findings suggest that while specialised training of experienced research staff could improve communication about the genomic methods of the research during the consent process, this will not necessarily be a topic of particular interest to individual participants. Participants may not be willing to spend additional time needed to understand the genomic nature of the research as opposed to those aspects of more interest, such as tangible risks and benefits of research participation. Previous experiences with the research institutions such as the NHRC and its researchers may also be important factors in decision-making, and have more influence on decision-making than information about a specific study. For example, we found that trust and the expectation of health benefits were important factors in the mothers’ decision to enrol their children in the MalariaGEN study.

Our data showed that participants’ parents understood the purpose of the MalariaGEN study as finding out why some children got severe malaria while others in similar environments didn’t. They were less familiar, however, with the methods that would be used to find this information, although they were aware that it would involve examining participants’ blood, and in some cases knew that it would be inheritable factors in the blood that make each person different that would be examined. Greater familiarity with the purpose of malaria research than the techniques that would be involved has also been found in rural Uganda [42] and Tekola and colleagues demonstrated similar levels of understanding of the genetic nature of research in rural Ethiopia [23].

A consequence of these levels of understanding is that participants will have little conception of the potential downstream uses of genomic data generated from their samples. As discussed in the results section, mothers expressed some support for the idea of reusing research samples in further studies. Similar findings were found in rural Uganda where 95% of participants in research agreed that further research could be conducted on linked samples with ethics committee permission, without returning to participants for further consent [42]. We believe that further research is needed to address the ethical issues arising in future uses of stored samples and getting a deeper understanding of the meanings behind participants’ responses to these issues.

Furthermore, some of us have argued elsewhere that a governed approach to data sharing is desirable and appropriate in response to the challenges in obtaining consent for genomic studies in Africa [17]. As the biomedical research enterprise increasingly incorporates complex scientific research methodologies, such as those involved in genomic research, there may be a need to focus more attention on additional protection mechanisms for research participants such as community engagement exercises and strengthening the review processes involved in these types of research projects.

### Limitations

This initial study aimed to identify issues arising when seeking consent to genetic and genomic research in a rural African context. As with other qualitative studies of this nature, the relevance of the findings to other research contexts requires further investigation, although a comparison with the published literature above suggests that some of the issues identified have relevance in other research contexts. It was beyond the scope of this study to include a comprehensive assessment of potential factors influencing understanding of genomic research in this population, such as low literacy levels and further research of this nature is likely to be valuable.

A specific limitation of this study is that it used questions about further research on identifiable blood samples as a proxy to begin exploring concerns about future research using genomic data. This is not ideal as the issues arising are related but not identical: some concerns raised by participants and fieldworkers had limited relevance to analysis of anonymised genomic data, such as linking potentially stigmatising results of novel research (including HIV status) to identifiable samples. Other issues that would require a familiarity with the complexities of analysis of anonymised genomic data were not expressed, such as the potential for such data to be stored and reused indefinitely.

Additionally, if not carefully thought through in advance, the phrasing of questions about blood samples could sometimes give the misleading and potentially upsetting impression that there will always be blood left over from the research. This may lead to concerns amongst parents about the amounts of blood taken for research and potential uses that may be made of ‘surplus’ blood. Following recent controversy about unanticipated genetic research being conducted with Havasupai samples in the US, further empirical research to determine attitudes to ongoing uses of samples and data from specific communities is desirable [43].

Finally, since this research was focused on the MalariaGEN project, we purposively recruited all the researchers, research assistants and research staff of the project in this study who gave consent for their inclusion in the study. The number of people in each category was limited by the number of researchers and research assistants working at the Navrongo Health Research Centre.

## Conclusions

Genomic research is a rapidly expanding field of research with which many researchers, field staff and ethical review committees have limited experience. For all these groups further education about the complex and sometimes novel ethical issues raised by such studies, and their implications for the design of appropriate methods for engaging with relevant research populations before, during and after genomic research will be necessary for the design and implementation of such research. This study demonstrates the importance of ensuring that resources are available for the design, conduct and evaluation of such education, even for research teams that have significant experience in other forms of healthcare research.

In light of the issues discussed above about difficulties arising when seeking consent to genomic research, we conclude that careful consideration needs to be given to additional protections for participants that may need to be implemented in such research, and how best to provide such protections. Capacity building for local ethics committees to enable appropriate consideration of ethical issues raised by genomic research is likely to be necessary in many contexts with limited experience of this form of research. Care will also be needed to determine the roles and responsibilities of local ethics committees and community representatives in contributing to the ongoing governance of genomic resources derived from their populations.

An additional important measure may be to ensure that community engagement activities are routinely conducted before, during and after, genomic research, as in the International HapMap Project. [44,45]. Even where the communities to be enrolled in research cannot be identified in advance, community engagement activities may provide a chance for researchers to consult about important aspects of genomic studies, including topics such as potential future uses of genomic data. Such activities can provide insights into community views on more complex and abstract aspects of research that, if appropriately documented, can inform both the design of consent processes and the decisions of community representatives and local ethics committees involved in ongoing governance of genomic resources. Further research is needed to determine how to appropriately engage with communities to elicit informed opinions of the acceptability of downstream uses of genome wide association data and other potential ethical issues.

## Abbreviations

FGD: Focus group discussion; GWA: Genome-wide association; KND: Kassena-Nankana District; NHRC: Navrongo Health Research Centre; VAST: Vitamin A supplementation trial (colloquial term for the NHRC).

## Competing interests

The authors have no competing interests. Two of the authors, PT and LAE, are married to each other. To avoid an appearance of competing interests, all of the research activities undertaken by PT were observed by or conducted in conjunction with one of the other authors (SB, JdV or RA). Where data collection by PT might be considered to raise concerns about competing interests (such as interviews conducted with staff supervised by LAE) such data collection was conducted by another author (JdV or RA).

## Authors' contributions

PT led the implementation of this project in Navrongo which involved tailoring the project to the local context, seeking ethics approval, identifying potential research participants, data collection, data analysis and developing the manuscript. SB designed and obtained funding for this project, as well as being integrally involved in analysis of the data and developing the manuscript. LAE was responsible for the implementation of the MalariaGEN study in Navrongo. He facilitated the conduct of this consent project and also provided substantial inputs to the manuscript. RA was involved in the implementation of this project in Navrongo which involved identifying potential research participants, data collection as well as preliminary data analysis and providing inputs to the manuscript. JdV supported the implementation of this project in Navrongo and made substantial contributions to the manuscript. KK is also an investigator on the MalariaGEN study in Navrongo and provided comments on the manuscript. DK is the Lead Investigator of MalariaGEN and provided comments on the manuscript. MP is a Principal Investigator on the MalariaGEN study and leads its ethics research programme. He has been involved in the project throughout and has provided comments on the manuscript. All authors read and approved the final manuscript.

## Pre-publication history

The pre-publication history for this paper can be accessed here:

http://www.biomedcentral.com/1472-6939/13/15/prepub
